# Quality of Life in Children With Achondroplasia Undergoing Paired Limb Lengthening With an External Fixator and Modified Distraction Control: Observational Nonrandomized Study

**DOI:** 10.2196/49261

**Published:** 2024-01-24

**Authors:** Vitaliy Trofimchuk, Bolatbek Dossanov, Vassiliy Lozovoy, Sergey Khmyzov, Assem Dossanova, Aleksandr Angelov, Andrey Pashenko, Olzhas Zhukenov

**Affiliations:** 1 Department of Pediatrician Surgery Non-Profit Joint Stock Company Astana Medical University Astana Kazakhstan; 2 Department of Pathology of the Spine and Joints of Children Sitenko Institute of Spine and Joint Pathology Kharkiv Ukraine

**Keywords:** achondroplasia, external fixator, quality of life, transosseous osteosynthesis, paired limb lengthening, bone growth disorder, dwarfism, limb lengthening, circular multiaxial system, hereditary disease, limb reconstruction, children, youth, pediatric, bone disorder, orthopedics, rehabilitation, bone, growth, disorder, genetic

## Abstract

**Background:**

Transosseous distraction osteosynthesis is prioritized in orthopedic care for children with achondroplasia. However, difficulties encountered during treatment and rehabilitation directly impact patients’ quality of life. Using rod external fixators within a semicircular frame for osteosynthesis is less traumatic compared to spoke circular devices. Their straightforward assembly and mounting on the limb segment can help significantly reduce treatment duration, thereby improving children’s quality of life during treatment and rehabilitation.

**Objective:**

This study aimed to conduct a comparative analysis of the quality of life (measured by postoperative pain syndrome, physical activity, and emotional state) among children with achondroplasia undergoing paired limb lengthening using either an external fixator with modified distraction control or a circular multiaxial system developed by the authors.

**Methods:**

This was an observational, prospective, nonrandomized, and longitudinal study with historical control. The study group consisted of 14 patients ranging from 5 to 15 (mean 7.6, SD 2.3) years old with a genetically confirmed diagnosis of achondroplasia. All patients underwent paired limb lengthening with a rod external fixator and a modified distraction control developed by the authors. A total of 28 limb segments, among them 4 (14%) humeri, 8 (29%) femurs, and 16 (57%) tibias, were lengthened in 1 round. Unpublished data from the previous study served as the control group, comprising 9 patients (18 limb segments) of the same age group (mean age at surgery 8.6, SD 2.3 years), who underwent limb lengthening surgery using a circular multiaxial system—2 (11%) humeri, 6 (33%) femurs, and 10 (56%) tibias. The Wong-Baker Faces Rating Scale was used to measure pain symptoms, while the Russified Pediatric Quality of Life (PedsQL) v4.0 questionnaire assessed quality of life.

**Results:**

During the latent phase (7 to 10 days after surgery), a more pronounced decrease in the indicators of physical activity and emotional state on the PedsQL v4.0 questionnaire was noted in the control group (mean 52.4, SD 4.8 versus mean 52.8, SD 5.5 points according to children’s responses and their parents’ responses, respectively) compared to the experimental group (mean 59.5, SD 6.8 points and mean 61.33, SD 6.5 points according to the children’s responses and their parents’ responses, respectively). The differences between the groups were statistically significant (*P*<.05 for children's responses and *P*<.01 for parents’ responses). Importantly, 6 months after surgery, these quality-of-life indicators, as reported by children in the experimental group, averaged 70.25 (SS 4.8) points. Similarly, their parents reported a mean of 70.54 (SD 4.2) points. In the control group, the corresponding values were 69.64 (SD 5.6) and 69.35 (SD 6.2), respectively. There was no statistically significant difference between the groups.

**Conclusions:**

The external fixator with modified distraction control developed by the authors provides a higher standard of living compared with the circular multiaxial system during the latency phase.

## Introduction

Achondroplasia is a hereditary disease characterized by a deceleration in bone and cartilage growth. The term “achondroplasia” was first used in 1878 by Jules Parrott, and in 1900, the neurologist Pierre Marie first described the main features of the disease in children and adults. According to the International Classification of Diseases (ICD-10), this pathology is classified in chapter XVII “Congenital malformations, deformations, and chromosomal abnormalities” (Q00-Q99), specifically in the section “Congenital malformations and deformations of the musculoskeletal system.” More specifically, it falls under code Q77, which encompasses osteochondrodysplasia with defects of growth of tubular bones and spine. Within this category, Q77.4 is specifically designated for achondroplasia. This congenital skeletal disorder in children belongs to the group of systemic dysplasias [1] and is associated with a defect in the zone of cartilage proliferation [2].

At birth, children in this nosological group display a proximal shortening of the upper and lower extremities, a relatively short and narrow trunk, trident-shaped hands, and macrocephaly with hypoplasia of the middle third of the face and a protruding forehead. Growth parameters at birth are usually slightly less than normal, but with age, there is a progressive lag from the normal values (total shortening of the limbs is especially pronounced in the upper arms and thighs). Infants with achondroplasia are most characterized by decreased muscle tone, causing them to learn movement and walking skills later in life. Intellect and cognitive abilities are not affected by this malformation [3,4]. A review of the specialized literature showed that the incidence of achondroplasia varies widely from 1:15,000 to 1:30,000 newborns, regardless of gender or race [5]. The main cause of achondroplasia is a de novo mutation in fibroblast growth factor receptor-3 (FGFR3), which leads to a disruption of the endochondral ossification mechanism [6].

Despite a wide array of pathological symptoms, disproportional dwarfism remains central in defining the stereotypes and lifestyle of patients living with this condition. It is characterized by significant limb shortening and deformity. The combination of external and radiological manifestations in the musculoskeletal system, which are exacerbated in the process of growth, strongly influences the way these patients perceive themselves and lead their lives. This issue is particularly marked in childhood, where more attention is paid to a person’s appearance [7,8].

Currently, transosseous distraction osteosynthesis is prioritized in orthopedic care [9,10]. This method is based on the general biological property of tissues to respond by regeneration to dosed stretching [11]. The conventional approach for uniform tubular bone lengthening typically involves 1 mm per day in 0.25 mm fractions across 4 sessions [12]. However, the period of osteosynthesis in this mode varies from 4 to 18 months, which correlates with the planned magnitude of lengthening [13,14]. Challenges encountered during treatment and rehabilitation significantly impact patients’ quality of life [15]. Traditionally, the Ilizarov circular system has been utilized for limb lengthening in patients in this nosological group [9]. The features of this equipment, as well as the fundamental studies on reparative tissue regeneration processes and the proposed surgical intervention options, remain highly relevant to this day. [16]. However, the complexity of the design, its excessively bulky nature, and its many parts can lead to long assembly times and require an increased time under anesthesia. In turn, these factors contribute to challenges during rehabilitation, limiting the use of this type of external fixator in pediatric practice [17]. Nevertheless, external fixators are the most common in the treatment of patients with achondroplasia in many countries [18-20]. According to the available literature, osteosynthesis with rod external fixators based on a semicircular frame is less traumatic compared to spoke circular devices. Moreover, rod fixators lead to less disruption of venous and lymphatic outflow in the postoperative period [20]. Rod fixators are more compact in appearance and provide sufficient rigidity to aid in bone fragment stabilization. Their straightforward assembly and mounting on the limb segment can help significantly reduce surgery duration, which is important in paired limb lengthening [21]. The authors developed a bar external fixation device with a distraction control system that showed better results than the circular multiaxial system regarding fixation time, regenerative length, deformation angles, pain intensity indexes, and complication rates [11]. This study aims to compare the quality of life (focusing on postoperative pain syndrome, physical activity, and emotional state) of children with achondroplasia undergoing paired limb lengthening using 2 different methods: an external fixator with modified distraction control and a circular multiaxial system developed by the authors.

## Methods

### Study Design

This was an observational, prospective, nonrandomized, and longitudinal study with a historical control. The experimental group included 14 patients, including 8 (57%) males and 6 (43%) females, aged between 5 and 15 (mean 7.6, SD 2.3) years. All patients had a genetically confirmed diagnosis of achondroplasia and received treatment at the state municipal enterprise “Multiprofile City Children's Hospital No 2” in Astana, Kazakhstan, spanning from August 2018 to January 2020. All patients underwent paired limb lengthening using a rod external fixator with modified distraction control developed by the authors. A total of 28 limb segments, including 4 (14%) humeri, 8 (29%) femurs, and 16 (57%) tibias, were lengthened in 1 round. All operations were performed by the same team of surgeons. The patients were dynamically followed up for 18 months.

Unpublished data from the previous study were used as the control group, which comprised 9 patients, including 3 (33%) males and 6 (67%) females, matching the same age group (mean age during surgery 8.6, SD 2.3 years). Patients in the control group also had a genetically confirmed diagnosis of achondroplasia and underwent limb lengthening surgery using a circular multiaxial system between January 2012 and July 2018. A total of 18 segments of tubular bone were lengthened in the control group—comprising 2 (11%) humeri, 6 (33%) femurs, and 10 (56%) tibias. All operations were performed by the same team of surgeons as in the experimental group. This study did not involve a clinical trial.

### Clinical Examination

Patients underwent preliminary clinical and radiological assessments. The clinical evaluation included orthopedic and neurological status: assessment of ligamentous elasticity and mobility of the knee joint, presence of torsional deformities of the lower extremities, child growth, and proportionality of the skeletal structure. The radial diagnostic protocol included radiographs of both lower extremities in straight projection over the entire length in a bipedal standing position with the correct orientation of the patellas (facing forward). Angular changes in the extremities were analyzed based on the radiographs obtained. The patients were examined by various specialists, including a pediatrician, endocrinologist, neurologist, cardiologist, and otolaryngologist, during the preoperative phase to identify any concomitant pathologies and mitigate intra- and postoperative complications.

### Operative Technique

**Figure 1:**
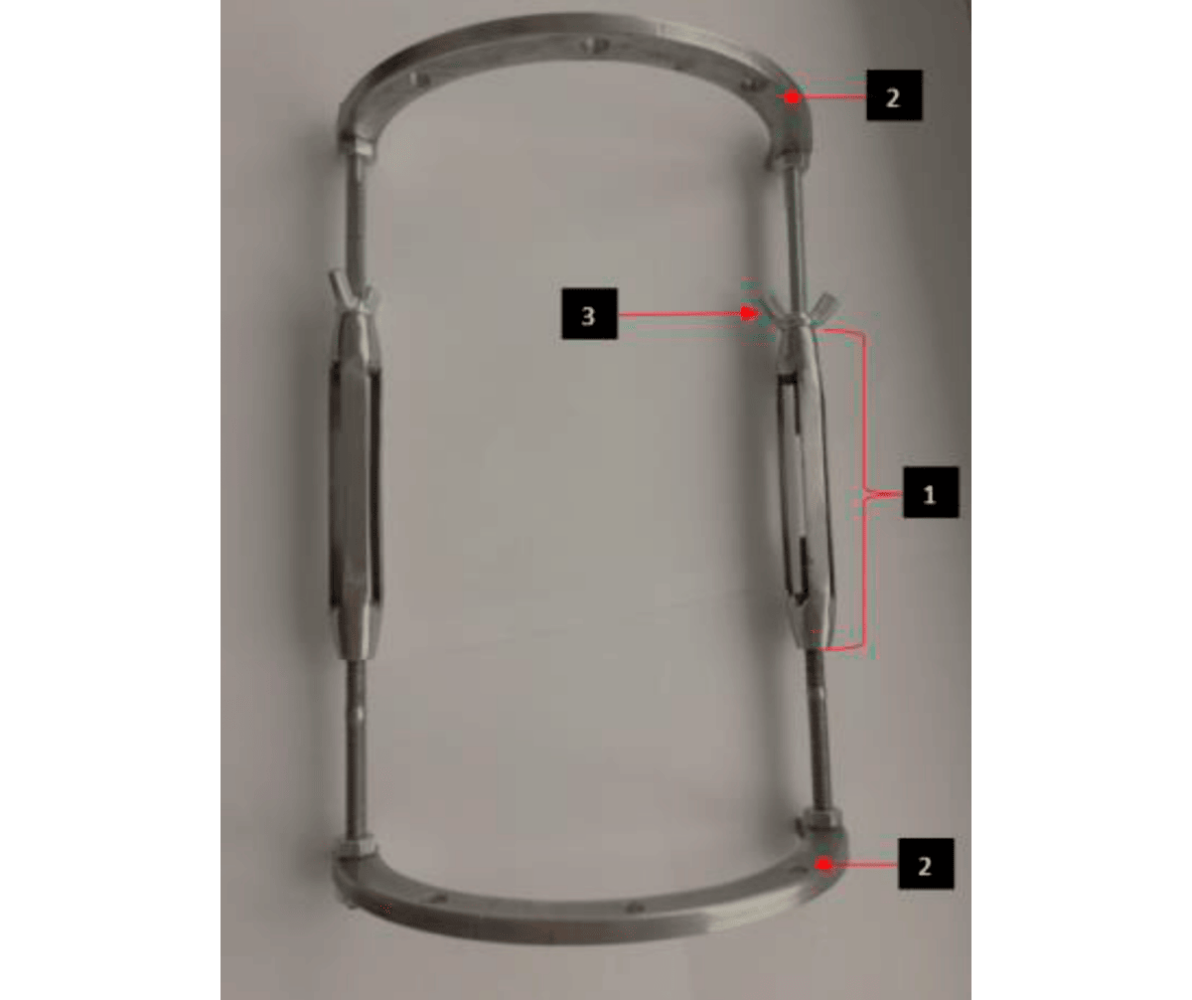
A semicircular external rod fixator design with the authors' modified distraction mechanism. (1) Mechanism of the fixator in the form of a 2-section sliding structure. External rod section with internal thread and 2 rods with an external millimeter thread. (2) Supporting bases on which the distraction system is fixed when installing an external fixation device on a limb segment. The 1-mm distraction step is performed by axial rotation according to the marks. (3) Nut stabilizing internally threaded rods on the proximal threaded rod.

Surgical treatment was performed under general endotracheal anesthesia. During the surgical procedure, a semicircular external rod fixator design with a distraction mechanism of the authors’ modification was used (Figure 1). The operations were performed simultaneously on 2 identical segments, according to the tibia-tibia and femur-femur schema. To minimize the traumatic nature of the surgical intervention, a closed corticotomy of the middle third of the diaphysis was performed.

### Postoperative Rehabilitation

Postoperative rehabilitation for patients with achondroplasia comprised 3 steps: a latency phase, a period of distraction and consolidation, and a period of functional adaptation of patients after device removal. The latency phase lasted 7 to 10 days, depending on the duration of postoperative edema recession and pain intensity. Lengthening was initiated at the end of the latent phase on the 7th to 10th day after surgery, with an average daily distraction rate of 0.75 mm. Restorative treatment was initiated on the second day after surgical intervention with constant parental involvement.

The amount of exercise depended on pain levels, distal limb swelling, and the patient’s psychological state. To prevent contractures of adjacent joints, the focus was on passive-active exercises ranging from 5 to 10 minutes, up to 3 times a day. Under medical supervision, patients were gradually mobilized to stand upright using walkers for up to 5 minutes and were taught to walk within the room. During distraction, the time of passive and active joint development sessions increased to 40 minutes, occurring 5 to 6 times a day, while the walking duration extended to 15 minutes.

The hospital stay for patients typically ranged from 10 to 14 days, adhering to the Republic of Kazakhstan’s Standard of Medical Care in Hospital Conditions. The hospital stay was determined based on the duration of the latency phase (period of postoperative edema recession and reduction of pain intensity). Subsequently, patients were discharged to outpatient treatment. Distraction and consolidation timing were assessed using radiographs. Control examinations with radiographs were performed every 10 days. During the examination, external fixator stability, joint function, and the presence of neurological and vascular disorders were evaluated. Based on the radiological appearance of the regenerate and assessment of joint mobility, the distraction rate was corrected (either decreased to 0.75 mm/day or increased to 2 mm/day). During the stabilization period, when performing joint development, an emphasis was placed on increasing muscle strength. Moreover, physical therapy classes remained intense, and the patients were taught to walk without additional support.

After reaching the possible segment length, the distraction process for the regenerate was halted, and the patients were examined monthly during the consolidation phase. After removing the fixators, a period of functional adaptation began that lasted up to 18 months after surgery. A key principle during this stage involved a gradual and appropriate increase in load. The treatment approach involved massaging the muscles of the thigh, lower leg, and humerus, coupled with physical therapy and thermal procedures. Furthermore, passive mobilization of all ranges of motion in the hip and knee joints was undertaken, with an emphasis on enhancing knee joint flexion. Patients were recommended to swim and exercise using simulators. Additionally, sanatorium-resort treatment was geared toward recovering all body systems following inpatient surgical treatment. Patients and parents were trained in the proper care of the medical device and rods and were instructed to adhere to the prescribed limb lengthening (distraction) schedule.

### Quality of Life Assessment

Postoperative pain is a complex response to tissue trauma during surgery. A pronounced postoperative pain syndrome increases the likelihood of postoperative complications, prolongs the patient’s recovery period and subsequent rehabilitation, reduces physical activity, and worsens the patient’s psychoemotional state. Postoperative pain intensity is determined not only by the extent of damage but also by psychological factors (accompanying emotional state and anxiety). In this regard, postoperative pain syndrome, physical activity, and patients’ emotional states were considered when assessing quality of life.

The Wong-Baker Faces Rating Scale was used to assess the pain syndrome [22]. When working with this rating scale, a child had to choose 1 of the 6 faces drawn that corresponded to how they felt. The first face represented 0 points and indicated “no pain,” while the sixth face represented 5 points and indicated “severe pain.” Pain was assessed in the latency and distraction phases.

To assess the quality of life, a questionnaire was administered using the Russified Pediatric Quality of Life (PedsQL) v4.0 questionnaire [23]. This questionnaire has 23 five-point scales reflecting the patients' current state: level of physical activity, emotional state, satisfaction with social role (satisfaction with communication with peers), and engagement in kindergarten/school. During this study, it was not feasible to correctly assess outcomes related to social role satisfaction and kindergarten/school attendance using the scales while the patients were still in the hospital. Therefore, quality of life was assessed only on the scales of level of physical activity and emotional state. The questionnaire consists of 2 parts: an assessment of a child's quality of life (from age 5 years) and an assessment of a child's quality of life by their legal representative. The children and their parents were instructed to select a number that reflected the frequency of difficult situations over a certain period, where 0 was never, 1 was almost never, 2 was sometimes, 3 was often, and 4 was almost always. The number of points was calculated by the questionnaire key. First, the results were reversed and converted to a linear 100-point scale, where 0 was 100, 1 was 75, 2 was 50, 3 was 25, and 4 was 0. Next, the survey results were tallied. The results of each item in the block were added up, and the resulting sum was divided by the number of items in the block. A score higher than 75 was considered optimal. In the third stage, the authors calculated the total score for each item and divided the result by the number of items. The questionnaire was administered in the preoperative, latency, distraction, and consolidation phase, as well as during dynamic follow-up (6, 12, and 18 months after surgery). The questionnaires were processed blindly.

### Statistical Analysis

The *t* test for the independent samples was used to assess the reliability of the differences between the experimental and control groups. The Student *t* test for dependent samples was also used to assess the reliability of differences within the groups at different stages of the study [24]. At *P*<.05, the null hypothesis of no relation between the parameters was rejected. Statistical calculations were performed using the SPSS software (IBM Corp).

### Ethical Considerations

The research was conducted in accordance with the Standard of Good Clinical Practices (GCP) to the Order of the Minister of Health and Social Protection of Kazakhstan (May 27, 2015; no 392) and the ethical standards of the Declaration of Helsinki, amended in 2013. Parents were informed in advance about the purpose of the planned surgery. Parents or legal guardians signed informed consent for the surgical intervention, rehabilitation treatment, and publication of the findings without identifying themselves. The study was reviewed and approved by the Human Research Ethical Committee of Astana Medical University (reference number 333).

## Results

In 9 (64%) patients in the experimental group, the lengthening results were evaluated as ”excellent.” This means that the planned elongation value had been reached, the deformation of the bone regenerate did not exceed 2 degrees, joint function was excellent (absence of contractures), and consolidation was successful based on radiographs. In 4 (29%) of patients, the lengthening results were evaluated as “good,” indicating the planned elongation value had been attained, with slight deformation of the bone regenerate (not exceeding 4 degrees), the presence of easily treatable contractures, and successful consolidation confirmed by radiographs. In 1 (7%) of cases, the results were classified as “satisfactory.” In these cases, the planned elongation was not fully achieved, there was some deformation of the bone regenerate (not exceeding 8 degrees), and there was a presence of contractures, but consolidation was successful according to radiographs.

Most patients achieved a lengthening value close to the planned value and correction of deformity, with minimal deviation that was not statistically significant. The average lengthening values were 8.5 (SD 0.6) cm, with the humerus length increasing by an average of 53% (SD 5%), the tibia by 52% (SD 8.2%), and the femur by 30% (SD 6%). The fixation period, including the distraction phase, averaged 83.8 (SD 3.7) days, with a specific average duration of 76 (SD 1) days for the humerus, 83.9 (SD 3.2) days for the tibia, and 87.5 (SD 2.5) days for the femur.

No contractures were observed during the latency phase or after the end of the distraction phase. However, during the distraction stage, 1 (7%) patient experienced knee joint contractures during hip lengthening, and 2 (14%) patients had ankle joint contractures due to heel tendon shortening, which resulted from failure to follow the treatment regime and joint development recommendations. The most common complaint reported by patients and their parents was minor inflammation of the soft tissues around the rods, which was resolved with conservative treatment. No cases necessitating rod removal or a second operation were noted. In the control group, the fixation time in the device averaged 101.4 (SD 5.4) days and the length of the regenerate averaged 6.6 (SD 0.8) cm. In 4 (29%) cases, knee joint contracture persisted, and 1 (7%) case of needle fracture was recorded.

Regarding pain, on the second day after the operation, the pain index in 13 (93%) patients in the experimental group was rated at 3 points on the Wong-Baker scale and at 4 points for 1 (7%) patient. However, by the end of the latency phase, the pain index in all patients was 0. In the control group, the Wong-Baker pain score was 4.1 (SD 1.02) on the second day and decreased to 1.7 (SD 0.8) at the end of the latency phase.

Before the surgery, quality of life scores on the PedsQL v4.0 questionnaire (measuring physical activity and emotional state) in the experimental group averaged 78.67 (SD 5) in the children's responses and 78.25 (SD 5.1) in their parents' responses. In the control group, these scores were 78.8 (SD 4.4) for the children and 78.0 (SD 5.4) for their parents. Thus, there were no differences in quality-of-life scores between the 2 groups before surgery.

As expected, during the latency phase following surgery, there was a significant decrease in physical activity and emotional state scores on the PedsQL v4.0 questionnaire in both groups when compared to the preoperative period. However, this decrease was more pronounced in the control group, with scores averaging 52.4 (SD 4.8) points by the children and 52.8 (SD 5.5) points by their parents. In contrast, in the experimental group, these quality-of-life scores decreased to 59.5 (SD 6.8) points according to the children's responses and 61.33 (SD 6.5) points according to their parents. These differences between the groups were statistically significant (*P<*.05 for the children's answers and *P*<.01 for their parents). At the same time, the experimental group showed a statistically more pronounced decline in the quality of life when the humerus was lengthened compared to the tibia and femur *(P<*.01). However, in the control group, such differences in quality-of-life changes between the lengthened segments were not observed.

By 6 months after surgery, there were improvements in physical activity and emotional state scores in both groups. These quality-of-life indicators on the PedsQL v4.0 questionnaire in the experimental group averaged 70.25 (SD 4.8) points according to the children's responses and 70.54 (SD 4.2) points according to their parents. In the control group, the corresponding scores were 69.64 (SD 5.6) points and 69.35 (SD 6.2) points, respectively. There was no statistically significant difference between the groups. There was also no difference between the lengthening segments in either group.

At 18 months after surgery, quality-of-life indicators (physical activity and emotional state scores) in both groups exceeded preoperative scores. In the experimental group, the average score was 84.3 (SD 2.5) group for the children and 85 (SD 2.5) points for their parents. These increases were statistically significant (*P<*.01). In the control group, the average score was 81.33 (SD 3.5) points for the children and 82.0 (SD 3.6) points for their parents, but the differences from preoperative scores were statistically unreliable. Furthermore, differences in quality-of-life scores between the experimental and control groups 18 months after surgery were statistically unreliable. The results of the PedsQL v4.0 quality of life questionnaire, completed by the patients and their parents in both groups, are shown in Tables 1 and 2.

**Table 1:** Results of transosseous osteosynthesis using the advanced rod monolateral external fixator and PedsQL^a^ v4.0 questionnaire scores completed by patients and their parents in the experimental group (N=14).

Gender	Age (years)	Segment	Consolidation period (days)	Planned lengthening (cm)	Lengthening results (cm)	PedSQL^a^ v4.0 questionnaire scores			
						Preoperatively	Latency phase (7-10 days after surgery)	6 months after surgery	18 months after surgery
Male	5	Tibia	82	10	Right: 8.3 Left: 8.5	Child: 72 Parent: 70	Child: 65 Parent: 68	Child: 68 Parent: 68	Child: 86 Parent: 86
Male	7	Tibia	85	10	Right: 8.9 Left: 8.4	Child: 78.3 Parent: 75	Child: 67 Parent: 66	Child: 66 Parent: 68	Child: 80 Parent: 83
Male	5	Tibia	85	10	Right: 7.9 Left: 8.2	Child: 80 Parent: 80	Child: 55 Parent: 55.3	Child: 66 Parent: 68	Child: 83 Parent: 86
Female	5	Tibia	79	10	Right: 8.3 Left: 8.3	Child: 78 Parent: 77	Child: 58 Parent: 57.3	Child: 66 Parent: 65.3	Child: 79.1 Parent: 80
Male	6	Tibia	80	10	Right: 10 Left: 10.2	Child: 87 Parent: 78.3	Child: 60 Parent: 62	Child: 68.3 Parent: 66	Child: 88.3 Parent: 86
Male	5	Tibia	88	10	Right: 9.1 Left: 8.9	Child: 75 Parent: 75	Child: 57 Parent: 57	Child: 66 Parent: 68.3	Child: 86 Parent: 85
Male	6	Tibia	87	10	Right: 10.3 Left: 9.9	Child: 77 Parent: 76	Child: 62 Parent: 65	Child: 65 Parent: 65	Child: 80 Parent: 78.3
Female	8	Tibia	85	10	Right: 8.2 Left: 8.5	Child: 80 Parent: 80	Child: 63 Parent: 64	Child: 72 Parent: 70	Child: 86 Parent: 85
Female	8	Femur	85	8.5	Right: 8.3 Left: 8.3	Child: 83 Parent: 84	Child: 60 Parent: 65	Child: 75 Parent: 75	Child: 86 Parent: 88.3
Female	12	Femur	90	8.5	Right: 7.2 Left: 7.2	Child: 82 Parent: 83	Child: 70 Parent: 72	Child: 78.3 Parent: 77	Child: 84 Parent: 84
Male	9	Femur	90	8.5	Right: 9 Left: 9	Child: 70 Parent: 71	Child: 52.3 Parent: 55	Child: 72 Parent: 70	Child: 78.3 Parent: 80
Male	6	Femur	85	8	Right: 8 Left: 8.2	Child: 80 Parent: 82	Child: 52 Parent: 56	Child: 80 Parent: 77	Child: 86 Parent: 88
Female	15	Humerus	75	9	Right: 7.5 Left: 7.8	Child: 85.3 Parent: 86	Child: 45 Parent: 50	Child: 75 Parent: 78.3	Child: 88.3 Parent: 87
Female	10	Humerus	77	8	Right: 8.1 Left: 8.2	Child: 80 Parent: 78.3	Child: 55.3 Parent: 56	Child: 72 Parent: 72	Child: 86 Parent: 86

^a^PedSQL: Pediatric Quality of Life.

**Table 2:** Results of transosseous osteosynthesis using the circular multiaxis system and PedsQL^a^ v4.0 questionnaire scores completed by patients and their parents in the control group (N=9).

Gender	Age (years)	Segment	Consolidation period (days)	Lengthening results (cm)	PedsQL^a^ v4.0 questionnaire scores			
					Preoperatively	Latency phase (7-10 days after surgery)	6 months after surgery	18 months after surgery
Female	7	Humerus	90	Right: 7 Left: 7	Child: 75 Parent: 74	Child: 52 Parent: 48	Child: 62 Parent: 57	Child: 86 Parent: 82
Female	6	Tibia	92	Right: 8 Left: 8	Child: 80 Parent: 75	Child: 57 Parent: 56	Child: 62 Parent: 66	Child: 82 Parent: 84
Female	7	Femur	105	Right: 8 Left: 8	Child: 80 Parent: 78	Child: 52 Parent: 56	Child: 66 Parent: 67	Child: 86 Parent: 86
Female	9	Femur	107	Right: 8 Left: 8	Child: 75 Parent: 73	Child: 58 Parent: 59	Child: 68 Parent: 66	Child: 80 Parent: 82
Male	8	Femur	102	Right: 6 Left: 6	Child: 88.3 Parent: 86	Child: 62 Parent: 60	Child: 67 Parent: 65	Child: 87 Parent: 88
Male	9	Tibia	95	Right: 6 Left: 6	Child: 83 Parent: 83	Child: 47 Parent: 52	Child: 68 Parent: 67	Child: 82 Parent: 83
Female	13	Femur	105	Right: 7 Left: 7	Child: 76 Parent: 76	Child: 56 Parent: 58	Child: 63 Parent: 65	Child: 78 Parent: 80
Female	14	Tibia	103	Right: 7 Left: 7	Child: 78.3 Parent: 78	Child: 46 Parent: 42	Child: 68 Parent: 66	Child: 80 Parent: 82
Female	14	Tibia	110	Right: 5 Left: 6	Child: 76 Parent: 72	Child: 56 Parent: 52	Child: 77 Parent: 75	Child: 78 Parent: 76
Male	7	Femur	107	Right: 5 Left: 5	Child: 76 Parent: 73	Child: 56 Parent: 58	Child: 76 Parent: 77	Child: 82 Parent: 84
Male	6	Tibia	95	Right: 6 Left: 6	Child: 73 Parent: 71	Child: 48 Parent: 49	Child: 72 Parent: 72	Child: 76 Parent: 80
Male	7	Femur	107	Right: 6 Left: 6	Child: 86 Parent: 85	Child: 56 Parent: 55	Child: 72 Parent: 76	Child: 85.3 Parent: 86
Female	6	Tibia	97	Right: 6 Left: 6.2	Child: 86 Parent: 86	Child: 46 Parent: 48	Child: 78 Parent: 80	Child: 79 Parent: 76
Female	7	Femur	105	Right: 6.5 Left: 6.5	Child: 80 Parent: 82	Child: 42 Parent: 46	Child: 76 Parent: 72	Child: 77.3 Parent: 79

^a^PedSQL: Pediatric Quality of Life.

**Figure 2:**
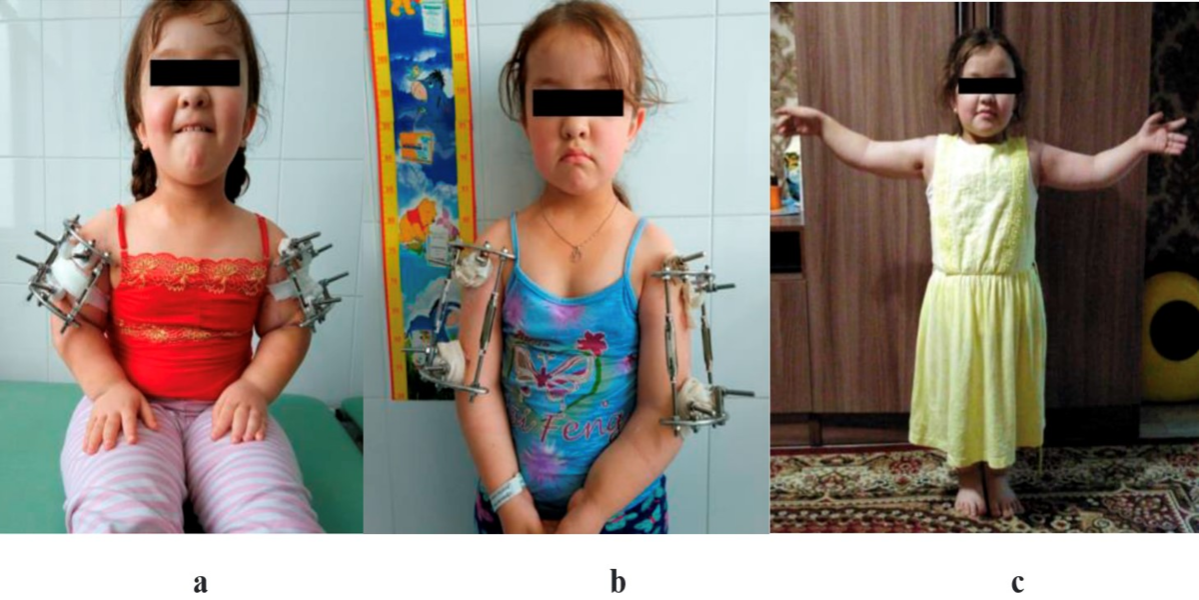
The postoperative dynamics of a 10-year-old patient diagnosed with achondroplasia who underwent paired limb lengthening with a rod external fixator and modified distraction control developed by the authors. (a) Patient 3 days after surgery (latent phase); (b) patient 3 months after surgery (consolidation phase); (c) progress 1 month after removal of the fixators (functional adaptation phase).

Figure 2a-c also shows the postoperative progression of a 10-year-old patient diagnosed with achondroplasia who underwent paired limb lengthening with a rod external fixator equipped with the authors’ modified distraction control. The patient and her parents reported a significant improvement in her quality of life after the surgical intervention and rehabilitation.

## Discussion

### Principal Findings

This study compared quality-of-life indicators (measured by postoperative pain syndrome, physical activity, and emotional state) in children with a genetically confirmed diagnosis of achondroplasia undergoing transosseous distraction osteosynthesis using 2 different external fixators systems: a rod system with the authors’ modified distraction control and a circular multiaxial system (Ilizarov system).

As expected, the results confirmed a decline in the quality of life for patients in both groups during the latency phase. However, patients in the control group (using the circular multiaxial system) experienced a more significant decrease in quality-of-life satisfaction, as reported by both the children and their parents/caregivers, compared to the experimental group using the rod fixator with the authors’ modified distraction control. Moreover, the control group reported more intense pain syndrome compared to the patients using the authors’ modified semicircular distraction system. During the later postoperative period under a dynamic observation, these differences decreased, and the level of satisfaction with the quality of life was statistically significantly higher in the main group 18 months after surgery than in the preoperative period.

Although orthopedic surgery for the treatment of achondroplasia has made significant advancements and continues to evolve, most practitioners have yet to agree on a surgical approach to the treatment of children and adolescents with this condition. Furthermore, the optimal fixator compositions for different age groups of patients are not specified [9]. A high rate of complications persists, which may be due to noncompliance with age-specific aspects of surgical treatment [17]. Several postoperative management issues remain unresolved [16].

In a recent study utilizing the PedsQL 4.0 questionnaire to assess the quality of life in children with achondroplasia (reported by the children and their parents/caregivers), it was observed that parents perceived their child’s quality to be lower in all domains compared to people of average height. This is due to physical limitations, barriers, and various challenges reported by children and adolescents to their parents. Notably, the children themselves also rated their quality of life significantly lower than the healthy control group, except in the emotional domain, where their scores were similar to the healthy group. It is possible that children with achondroplasia have learned to accept themselves as they are and find contentment despite experiencing significant physical limitations in their quality of life, both in school and social contexts [7]. It is important to understand that the diagnosis of achondroplasia and its consequences impact not only a child but also the entire family, as family members must adapt to the unique needs of the child [7].

Surveys conducted among patients with achondroplasia and their family members, both before and after treatment, consistently answer in favor of the need for limb augmentation [8,17]. Currently, the primary method for addressing growth deficit in patients with achondroplasia involves surgical distraction osteosynthesis [9,10]. The possibility of drug-assisted limb lengthening, particularly with the drug Vosoritide, is being studied. While the results are encouraging, at present, this trend cannot serve as an alternative to surgical treatment [4].

During surgical treatment, transosseous osteosynthesis is the most commonly used method, involving the use of external bone-anchored supports placed above the skin’s surface. However, patients are required to wear these systems throughout the distraction and consolidation period of the regenerate, which can last up to 18 months, depending on the planned degree of limb lengthening. This inevitably impacts a patient's quality of life. In response to this concern, internal fixation systems have been developed, such as the Precice system with magnetic control over distraction speed [25,26], and combined systems like LON (Lengthening Over Nail) and LATN (Lengthening and Then Nailing), which halve the time of fixator use [27-29]. However, these systems cannot always serve as an alternative to fixators because they use expensive titanium rods. The Precice system has limitations in bone diameter, cannot be used for humerus lengthening, and the procedure itself must be well planned since no postoperative changes (other than distraction rate) can be made [27]. The LON and LATN systems require additional surgical intervention. Consequently, the development of lighter and more comfortable fixators remains urgent.

Traditionally, limb lengthening for patients in this nosological group has been performed using a multiaxial system, known as the Ilizarov system. While this system shows good results in reparative tissue regeneration processes, its complex design and cumbersomeness can impact patients’ quality of life, which is especially significant in pediatric practice [9,16,17]. To address this, rod fixators built on a semicircular frame with a simpler and lighter design are gaining popularity [20,21]. The authors have introduced a rod fixator with modified distraction control. A previous article demonstrated the advantage of this system over the circular multiaxial system, highlighting improvements in fixation time, achieved regenerative length, correction of deformities, pain intensity, and complication rates [11].

This study establishes that the authors’ rod fixation with modified distraction control facilitates an improved standard of living compared to a circular multiaxial system in the latent phase. Consequently, this advancement not only allows patients with achondroplasia to move freely from the first days after surgery but also to gradually develop strength in the lengthened limb.

### Conclusions

The rod fixator with modified distraction control developed by the authors significantly enhances the quality of life compared to the circular multiaxial system in the latency phase. Employing this fixator technique for paired surgical lengthening in children with achondroplasia ensures stability throughout the distraction process, provides a strong and uniform regenerate, contributes to a significant reduction in complications, and allows patients to regain full physical activities in a shorter time. With its high stability, the device creates favorable conditions for psychological and physical adaptation during treatment and demonstrates a significant advantage over the circular multiaxial system. Considering the cost-effectiveness of this developed fixation system, it can contribute to delivering quality orthopedic care for patients with achondroplasia.

## Abbreviations

ICD: International Classification of Diseases
FGFR3: fibroblast growth factor receptor-3
GCP: Good Clinical Practice
LON: Lengthening Over Nail
LATN: Lengthening and Then Nailing
PedsQL: Pediatric Quality of Life

## Data Availability

The data sets generated and/or analyzed during this study are available from the corresponding authors upon reasonable request.
